# Evolution of therapeutic paradigms in Hodgkin lymphoma: from “cure-oriented” to “functional cure”

**DOI:** 10.3389/fonc.2026.1855868

**Published:** 2026-05-29

**Authors:** Chengqian Chen, Wei Guo, Xiaoyu Song, Xin Wan, Zhaoxia Li, Haotian Wang, Ou Bai

**Affiliations:** 1 Department of Hematology, The First Hospital of Jilin University, Changchun, Jilin, China; 2 Department of Endocrinology and Metabolism, The First Hospital of Jilin University, Changchun, China

**Keywords:** brentuximab vedotin, ctDNA, functional cure, Hodgkin lymphoma, PD-1 inhibitors, precision therapy, tumour microenvironment

## Abstract

Hodgkin lymphoma (HL) is a B-cell–derived hematologic malignancy originating from germinal centre B cells. Owing to its remarkable sensitivity to chemotherapy and radiotherapy, HL has become one of the most curable adult malignancies. Over the past decades, treatment regimens such as ABVD and escalated BEACOPP, combined with radiotherapy, have achieved 5-year survival rates exceeding 90% in early-stage patients and long-term remission rates above 75% in advanced-stage disease. However, long-term toxicities associated with conventional therapies—including secondary malignancies, cardiovascular disease, pulmonary fibrosis, and infertility—have increasingly compromised survivors’ quality of life. Meanwhile, approximately 5%–10% of patients exhibit primary refractory disease, and 10%–30% eventually relapse.In recent years, the emergence of targeted and immunotherapeutic agents, particularly the anti-CD30 antibody–drug conjugate (brentuximab vedotin) and immune checkpoint inhibitors (e.g., PD-1 inhibitors), has profoundly reshaped the therapeutic landscape of HL. This review systematically summarises the epidemiological characteristics and biological foundations of HL, as well as the achievements and limitations of conventional therapies. We place particular emphasis on key clinical trial evidence in recent years and discuss paradigm shifts in four major areas: treatment de-escalation and toxicity reduction in frontline therapy, precision salvage strategies for relapsed/refractory HL, the evolving role of transplantation, and the application of precision medicine tools such as circulating tumour DNA (ctDNA) and molecular subtyping. We further explore how HL treatment is transitioning from a “cure-oriented” model focused solely on survival to a novel paradigm of “functional cure” that balances efficacy with long-term quality of life. We define “functional cure” as durable remission with minimal long−term toxicity, preserving organ function and quality of life—a unifying principle of modern HL management. Special attention is given to clinical decision-making in PD-1–exposed patients, emerging therapeutic targets, cellular therapies, and the growing role of real-world evidence. These insights aim to inform future individualised treatment strategies in HL.

## 1 Introduction

Classical Hodgkin lymphoma (cHL) is a unique hematologic malignancy characterised by distinctive pathological features and a high degree of treatment sensitivity. From a global epidemiological perspective, cHL accounts for approximately 90% of all Hodgkin lymphoma cases, while HL itself represents about 10% of all lymphomas (1). According to GLOBOCAN 2020 data from the International Agency for Research on Cancer (IARC), the global age-standardised incidence and mortality rates of HL are 0.98 and 0.26 per 100,000 population, respectively, corresponding to approximately 83,000 new cases and 23,000 deaths annually (2). HL demonstrates a characteristic bimodal age distribution, with a first peak among adolescents and young adults (15–30 years) and a second smaller peak in individuals aged over 55 years.Males are slightly more affected than females overall,and the disease burden varies significantly across geographic regions (2–4). For instance, in the United States, an estimated 8,720 new cases and 1,150 deaths are expected in 2025 (3). whereas in China, the age-standardised incidence rate is substantially lower (approximately 0.23 per 100,000) (5), with a marked decline in mortality and disability-adjusted life years (DALYs) from 1990 to 2021, reflecting improvements in diagnosis and treatment (5).

Since its first description in 1832, the aetiology of HL remains incompletely understood. Nevertheless, cHL has become a paradigmatic success in oncology, with cure rates approaching 90% across disease stages(reviewed in (6)). These achievements stem from decades of optimisation in chemotherapy regimens, advances in radiotherapy techniques, and the development of risk-adapted treatment strategies. Due to excellent cure rates with conventional therapies, contemporary trials incorporate novel agents, including immune checkpoint inhibitors and targeted chemoimmunotherapy (brentuximab vedotin), with the goal of maintaining high cure rates while decreasing the risk of chronic toxicities such as secondary malignancies, cardiovascular disease, pulmonary fibrosis, and infertility, which compromise survivor quality of life. These toxicities affect multiple organ systems. In early-stage patients, long-term cardiovascular risk is particularly significant—cardiovascular mortality surpasses HL-related mortality approximately 5–10 years after diagnosis (7), pulmonary toxicity is also notable, with bleomycin-induced lung injury occurring in 6.5%–11% of patients (up to 21% in older individuals), and a markedly increased risk of death from interstitial lung disease (8). Reproductive toxicity is especially impactful and depends on cumulative alkylating agent exposure. In male survivors receiving high−dose alkylator−based regimens, post−treatment azoospermia has been reported in up to 33%–100% of cases (median 75%), whereas modern reduced−alkylator regimens carry lower risks (9); in females, premature ovarian insufficiency occurs in 6%–34% of patients, particularly following pelvic irradiation or high-dose chemotherapy(reviewed in (9)). Historically, with conventional chemotherapy, approximately 5%–10% of patients were refractory to first−line therapy, and 10%–30% relapsed after achieving complete remission (1). These dual challenges—long-term toxicity and treatment failure—have shifted the central clinical question toward how to maintain high cure rates while minimising treatment-related morbidity.

The advent of novel agents, particularly the anti−CD30 antibody–drug conjugate (brentuximab vedotin) and immune checkpoint inhibitors (e.g., programmed cell death‑1 (PD‑1) inhibitors), has provided a promising solution (10, 11). These therapies have demonstrated unprecedented efficacy in relapsed/refractory disease and have been successfully incorporated into frontline treatment. As a result, the concept of “functional cure”—maximising quality of life while preserving therapeutic efficacy—has transitioned from theoretical aspiration to clinical reality. This paradigm shift is further supported by landmark trials such as SWOG S1826 and GHSG HD21 (2024–2025), which established immunotherapy-based regimens as a new standard in frontline treatment, enabling over 90% of newly diagnosed patients to achieve durable disease-free survival (10, 11).

However, the widespread adoption of novel therapies has introduced new challenges. Key clinical questions now include: how to manage patients who relapse after PD-1 inhibitor exposure, how to implement biomarker-driven precision stratification, and which patients may safely omit transplantation. In this review, we systematically examine the epidemiological and biological foundations of HL, critically evaluate the achievements and limitations of conventional therapies, and highlight key clinical evidence underpinning the ongoing paradigm shift.

## 2 Biological basis and therapeutic targets of Hodgkin lymphoma

The unique pathological hallmark of cHL—a sparse population of malignant Hodgkin and Reed–Sternberg (HRS) cells embedded within a highly abundant and heterogeneous immune microenvironment—underpins its distinct pathogenesis, immune evasion strategies, and therapeutic vulnerabilities. A comprehensive understanding of HRS cell biology, aberrant signalling pathways, tumour microenvironment (TME) remodelling, and emerging molecular subtypes is essential for interpreting the ongoing evolution of therapeutic paradigms in HL. This section provides a systematic overview of these dimensions and their implications for targeted and immunotherapeutic strategies.

### 2.1 Characteristics and origin of tumour cells: HRS cells

Classical Hodgkin lymphoma is pathologically characterised by the presence of malignant HRS cells, which typically constitute less than 5% of the total tumour cellularity(reviewed in (12)). Morphologically, HRS cells are distinguished by abundant cytoplasm and characteristic bilobed nuclei with prominent eosinophilic nucleoli, often described as having an “owl’s eye” appearance (13).

It is now well established that HRS cells originate from germinal centre B cells (14). This conclusion is supported by the presence of clonal immunoglobulin gene rearrangements and somatic hypermutation patterns typical of germinal centre reactions. However, in contrast to normal B cells, HRS cells exhibit a profound loss of the canonical B-cell transcriptional programme, including the inability to express functional immunoglobulins, while retaining weak expression of the B-cell transcription factor PAX5 (14, 15). This phenomenon of “phenotypic reprogramming” is central to their aberrant biology.

From a therapeutic perspective, HRS cells almost uniformly express CD30, a member of the tumour necrosis factor receptor superfamily, whereas its expression in normal tissues is highly restricted, making CD30 an ideal therapeutic target (12, 16, 17). Immunophenotypically, HRS cells characteristically express CD15, while classical B-cell markers such as CD20 and the leukocyte common antigen CD45 are typically absent (12). It should be noted that the immunophenotype described above is specific to cHL. Nodular lymphocyte−predominant Hodgkin lymphoma (NLPHL) is a distinct entity, characterized by lymphocyte−predominant cells with the following markers: CD20^+^, CD45^+^, CD30^-^, and CD15^-^.

### 2.2 Aberrant activation of key signalling pathways

The survival and proliferation of HRS cells depend on the constitutive activation of multiple oncogenic signalling pathways. The NF-κB pathway represents the central survival axis in HRS cells. Its persistent activation arises from diverse mechanisms. Genetically, approximately 40% of cases harbour inactivating mutations or deletions of TNFAIP3 (encoding A20), leading to impaired negative feedback regulation of NF-κB signalling (18). In Epstein–Barr virus (EBV)-positive cases, the viral latent membrane protein 1 (LMP1) mimics CD40 signalling, resulting in constitutive NF-κB activation (19). Additionally, signals from the tumour microenvironment—including CD30 ligand, CD40 ligand, and BAFF—further sustain pathway activation (20). Activated NF-κB promotes the expression of anti-apoptotic proteins such as BCL-xL, thereby preventing apoptosis of defective B cells and supporting HRS cell expansion (21).

The JAK–STAT pathway is aberrantly activated in approximately 87% of cHL cases (22). A key driver is the amplification of chromosome 9p24.1, which encompasses PD-L1, PD-L2, and often JAK2. Co-amplification of JAK2 enhances pathway signalling, which in turn further upregulates PD-L1 expression due to its JAK–STAT–responsive promoter (23). Additional contributing alterations include loss-of-function mutations in SOCS1 (~60%) and gain-of-function mutations in STAT6 (~30%) (22). Beyond genetic drivers, autocrine cytokine signalling (e.g. IL-13, IL-21) and paracrine inputs from the microenvironment further reinforce pathway activation (14). Activated STAT3, STAT5, and STAT6 collectively promote HRS cell survival, proliferation, and transcriptional reprogramming (14).

The PI3K/AKT pathway is also constitutively active in most cHL cases, as evidenced by widespread AKT phosphorylation (24). Functional studies demonstrate that inhibition of PI3K/AKT signalling induces cell cycle arrest and apoptosis in HL cell lines, highlighting its essential role (25). Multiple mechanisms contribute to this activation, including receptor-mediated signalling (via CD30, CD40, RANK, and S1PR1) (25, 26); as well as genetic alterations such as recurrent mutations in GNA13 and ITPKB, both of which normally restrain PI3K/AKT signalling (19). Downstream, activated AKT suppresses FOXO1, a tumour suppressor transcription factor, thereby facilitating immune evasion and unchecked proliferation (27).

### 2.3 Tumour microenvironment and immune evasion

A defining feature of cHL is the striking imbalance between malignant cells and the surrounding microenvironment. HRS cells typically account for only 1%–5% of tumour cells, whereas the bulk consists of a diverse infiltrate of non-malignant immune and stromal cells, including T cells, B cells, macrophages, eosinophils, plasma cells, and fibroblasts (28). HRS cells actively shape this microenvironment by secreting a wide array of cytokines and chemokines, thereby establishing a niche that promotes tumour survival and suppresses anti-tumour immunity (28, 29).

The PD-1/PD-L1 axis represents the central mechanism of immune evasion in cHL (30). HRS cells overexpress PD-L1 and PD-L2, primarily driven by 9p24.1 amplification and JAK–STAT signalling activation (31). Engagement of PD-1 on tumour-infiltrating lymphocytes leads to T-cell exhaustion, characterised by impaired proliferation, cytokine production, and cytotoxic function, thereby allowing tumour immune escape (32). EBV-positive cHL exhibits distinct immune evasion features. These tumours typically have a lower tumour mutational burden, suggesting that viral oncogenic signalling may partially substitute for genetic alterations (33, 34), EBV-encoded proteins can independently upregulate PD-L1 expression, reinforcing immune escape mechanisms (33, 34).

Beyond PD-1 signalling, HRS cells orchestrate a broader immunosuppressive network. The secretion of thymus and activation-regulated chemokine (TARC/CCL17) recruits CCR4-positive CD4^+^ T cells and regulatory T cells, forming the characteristic “T-cell rosette” structure surrounding HRS cells (35, 36). Clinically, serum TARC levels correlate with tumour burden and treatment response, highlighting its role as a dynamic biomarker (35–37).

The CTLA-4/CD86 axis has also emerged as an additional immune checkpoint pathway (38). CD86 is highly expressed not only on HRS cells but also on tumour-associated macrophages, enabling coordinated inhibitory signalling to T cells via CTLA-4 (38). Although CTLA-4 blockade alone has shown limited efficacy in PD-1–exposed patients (38), this pathway provides further insight into the multidimensional immunosuppressive landscape of cHL. Notably, preclinical studies suggest that CD86-targeted CAR-T therapies may simultaneously eliminate HRS cells and immunosuppressive macrophages, offering a promising future strategy (39).

### 2.4 Advances in ctDNA-based molecular subtyping

Traditional histopathological classification of cHL provides limited guidance for therapeutic decision-making. In contrast, ctDNA–based molecular profiling has recently enabled significant advances in non-invasive tumour characterisation. Despite their low abundance in tumour tissue, HRS cells release disproportionately high levels of DNA into the circulation (40). Mechanistically, high expression of DNASE1L3 in the tumour microenvironment may facilitate DNA fragmentation and release, resulting in ctDNA levels that can exceed those observed in lymphomas composed predominantly of malignant cells (40). Comparative studies have demonstrated that ctDNA analysis can detect a greater number of tumour-specific mutations, often at higher variant allele frequencies, than tissue biopsy (41).

Based on ctDNA profiling, two principal molecular subtypes have been proposed. The C1 subtype (approximately 64%) is characterised by a high mutational burden, enriched for activation-induced cytidine deaminase (AID)-related signatures and microsatellite instability, and is more common in younger patients. In contrast, the C2 subtype (approximately 36%) is dominated by chromosomal instability, with frequent somatic copy number alterations, particularly amplification of 9p24.1 (encompassing PD-L1, PD-L2, and JAK2) (42). Additional classification frameworks have further resolved three biologically distinct subtypes, comprising an inflammatory immune-escape subtype characterized by 9p24.1 amplification and an inflamed microenvironment, a virus-driven subtype associated with EBV infection and low mutational burden, and an oncogene-driven subtype defined by high mutational burden along with recurrent mutations in genes such as TNFAIP3 and SOCS1 (43). These molecular classifications provide a biological basis for heterogeneity in treatment response, particularly to immunotherapy (43).

Notably, recent studies have highlighted the importance of non-coding mutations in cHL. Approximately 86% of patients harbour mutations in regulatory regions, including 30% with mutations affecting BCL6 expression quantitative trait loci, which enhance tumour proliferation (42). Furthermore, ctDNA-based analyses suggest that subclonal neoantigens are associated with shorter progression-free survival following PD-1 blockade, whereas clonal neoantigens predict more favourable outcomes, indicating that neoantigen clonality may serve as a predictive biomarker (42).

Collectively, ctDNA-based molecular profiling is driving a transition from traditional clinical risk stratification toward biologically informed classification. Ongoing prospective validation studies are expected to further establish its role in guiding treatment de-escalation and targeted therapeutic selection in cHL.

## 3 Evolution of treatment strategies in newly diagnosed Hodgkin lymphoma

The therapeutic goal in newly diagnosed HL has shifted from a singular focus on maximising cure rates to a more nuanced strategy that seeks to preserve efficacy while minimising both acute and long-term toxicity. This transition reflects the growing recognition that long-term survivors—often diagnosed at a young age—face substantial risks of treatment-related morbidity. Contemporary treatment strategies are therefore increasingly risk-adapted and biology-informed, with distinct approaches emerging according to disease stage and patient characteristics. (**Table 1**: Frontline Therapy/Newly Diagnosed HL).

**Table 1:** Frontline therapy/newly diagnosed HL.

Trial/study	Patient population	Age/stage	Regimen	Phase		Follow-up duration	Key endpoint(s)	Outcome	Reference
NIVAHL	Early-stage unfavorable HL	Pediatric & Adult	Nivolumab + chemo (concomitant or sequential) followed by 30 Gy IS-RT		II	3 years	OS, PFS	OS 100%; PFS 98–100% (concomitant: 100%, sequential: 98%)	(51)
BREACH	Early-stage unfavorable HL	Adults	BV + AVD vs ABVD		II	2 years	PET negativity after 2 cycles, PFS, OS	PET-negative rate higher for BV-AVD vs ABVD (82.3% vs 75.4%); 2-year PFS slightly higher for BV-AVD (97.3% vs 92.6%); high TMTV associated with shorter PFS	(54)
AHOD2131	Newly diagnosed stage I–II HL	Pediatric & Adult	Standard chemo ± RT vs BV + Nivolumab		III	up to 12 years (primary 3-year PFS)	PFS, OS	Ongoing; primary year aim: PFS comparison	(53)
SWOG S1826	Newly diagnosed stage III–IV cHL	Adolescents & Adults	N-AVD vs BV-AVD		III	3 years	PFS, OS	3-year PFS 91% vs 82%; HR 0.48; OS not reached	(55)
GHSG HD21	Newly diagnosed advanced-stage cHL	Adults	BrECADD vs escalated BEACOPP		III	5 years	PFS, OS	5-year PFS 93.6% vs 90.6%; OS 98% (both arms)	(57, 58)
ECHELON-1	Newly diagnosed stage III–IV HL	Adults	BV-AVD vs ABVD		III	6 years	PFS, OS	6-year PFS 82.3% vs 74.5%;6-year OS 93.9% vs 89.4%	(60)

### 3.1 Early-stage Hodgkin lymphoma: de-escalation while preserving efficacy

Early-stage HL is associated with an excellent prognosis, with traditional combined-modality therapy (2–4 cycles of chemotherapy followed by involved-field radiotherapy) achieving 5-year survival rates exceeding 90% (44). However, the long-term risks associated with radiotherapy—particularly secondary malignancies and cardiovascular disease—have made treatment de-escalation, especially radiotherapy omission, a central clinical objective (44, 45).

PET-adapted strategies have established the foundation for modern de-escalation approaches. Leveraging the high negative predictive value of interim PET/CT, several randomised trials have evaluated the feasibility of omitting radiotherapy in patients achieving early metabolic response (46, 47). The RAPID and H10 trials consistently demonstrated that, among patients with negative interim PET (Deauville score 1–3) after 2–3 cycles of ABVD, omission of radiotherapy resulted in only a modest absolute reduction in progression-free survival (approximately 3–5%), without compromising overall survival. Importantly, most relapses remained highly salvageable (46–49). Notably, pooled analyses revealed a predictable pattern of relapse: over 80% of recurrences occurred at initially involved sites and were concentrated within the first two years following treatment (50). This observation not only supports the safety of radiotherapy omission in selected patients but also informs surveillance strategies and salvage treatment planning. Consequently, PET-guided de-escalation has become a standard paradigm in early-stage HL.

The incorporation of novel agents is further reshaping this landscape. The NIVAHL trial represents the first prospective study evaluating PD-1 blockade in combination with chemotherapy in early-stage unfavourable HL (51). With both concurrent and sequential nivolumab-based regimens achieving near-perfect outcomes—100% overall survival and progression-free survival approaching 100% at 41 months—this study demonstrates the transformative potential of immunotherapy in frontline settings (51). Importantly, translational analyses revealed rapid molecular responses, including early clearance of ctDNA and a marked decline in serum TARC levels, suggesting that deep remissions may be achievable without reliance on radiotherapy (52). This cross-age collaborative principle has been extended to the ongoing Children’s Oncology Group phase III trial AHOD2131, which enrolls patients aged 5–60 years with newly diagnosed stage I/II cHL to evaluate brentuximab vedotin plus nivolumab in a response-adapted design, thereby harmonizing immunotherapy-based treatment across the age continuum (53).

Similarly, the BREACH trial evaluated brentuximab vedotin (BV) combined with AVD chemotherapy. While overall differences in progression-free survival compared with ABVD did not reach statistical significance, BV-AVD demonstrated higher early PET negativity rates and notable benefits in high-risk subgroups, particularly those with elevated baseline metabolic tumour volume (54).

Taken together, early-stage HL management is undergoing a paradigm shift. PET-adapted de-escalation has become the standard backbone, while the integration of PD-1 inhibitors and BV is enabling deeper remissions with potentially reduced reliance on radiotherapy. These advances collectively support the emerging goal of achieving durable disease control with minimal long-term toxicity—hallmarks of a “functional cure”.

### 3.2 Advanced-stage Hodgkin lymphoma: redefining efficacy and toxicity profiles

Advanced-stage HL has become the primary arena for therapeutic innovation and represents the clearest realisation of the “functional cure” paradigm. Recent landmark trials have fundamentally redefined treatment standards.

The SWOG S1826 trial, a large randomised phase III study, directly compared nivolumab plus AVD (N-AVD) with BV-AVD in newly diagnosed stage III–IV cHL (10). Updated 3-year results demonstrated a significant improvement in progression-free survival with N-AVD (91% vs 82%; HR 0.48), with consistent benefit across all predefined subgroups (55). Notably, this regimen achieved excellent outcomes in adolescent populations while markedly reducing the need for consolidative radiotherapy. Of note, the SWOG S1826 trial enrolled both adolescents and adults, reflecting a growing trend of cooperative group trials that bridge pediatric and adult populations. These findings have established N-AVD as a new frontline standard and prompted updates in international guidelines (56).

In parallel, the GHSG HD21 trial exemplifies an alternative strategy—optimising chemotherapy backbones to reduce toxicity without compromising efficacy. The BrECADD regimen, which replaces bleomycin and procarbazine with less toxic agents, demonstrated superior 5-year progression-free survival compared with escalated BEACOPP (93.6% vs 90.6%) while maintaining overall survival rates approaching 98% (57, 58). Crucially, HD21 provides some of the most compelling evidence for toxicity reduction. BrECADD significantly improved gonadal function recovery in both males and females, reduced the incidence of peripheral neuropathy, and lowered the risk of secondary malignancies. These findings highlight that treatment optimisation is no longer limited to survival outcomes but extends to long-term functional preservation (11, 58, 59).

Long-term follow-up of the ECHELON-1 trial further supports the role of BV-AVD, demonstrating sustained improvements in both progression-free and overall survival at six years, particularly among high-risk patients (60). Although N-AVD has emerged as the preferred regimen, BV-AVD remains a valid alternative in selected populations.

In summary, advanced-stage HL treatment has entered a new era defined by dual optimisation of efficacy and toxicity. Immunotherapy-based regimens such as N-AVD establish new efficacy benchmarks, while chemotherapy refinements such as BrECADD demonstrate that long-term toxicity can be meaningfully reduced without sacrificing cure rates. Together, these approaches provide the strongest clinical evidence to date supporting the feasibility of “functional cure”.

### 3.3 Treatment advances in elderly patients with Hodgkin lymphoma

Elderly patients (typically defined as ≥60 years) account for approximately 20%–25% of HL cases but experience significantly worse outcomes compared with younger populations (61). This disparity reflects a combination of factors, including increased comorbidity burden, reduced functional reserve, and poor tolerance to conventional chemotherapy (61–63).

The N-AVD regimen has emerged as a preferred frontline option in older patients with advanced disease. Subgroup analyses from the SWOG S1826 trial demonstrated substantial improvements in both progression-free survival (89% vs 64% at 2 years) and overall survival (96% vs 85%) compared with BV-AVD (64). Importantly, despite higher rates of neutropenia, N-AVD was associated with fewer treatment discontinuations and fewer infection-related complications (64). Updated 3-year follow-up data further confirmed the durability of these benefits, with progression-free survival (PFS) rates of 82% and 58% in older patients, who derived the most pronounced advantage from N-AVD (55).

The BrECADD regimen has also shown promising efficacy in selected elderly patients. A prospective cohort study in patients aged 61–75 years reported a 2-year progression-free survival of 91.5% and overall survival of 90.8%, with no treatment-related mortality (59). Notably, quality-of-life measures returned to baseline levels following treatment, supporting the feasibility of this approach in carefully selected individuals.

Real-world data further reinforce the value of novel agents in elderly populations. A large multicentre retrospective study demonstrated that regimens incorporating BV and/or PD-1 inhibitors significantly improved survival compared with conventional chemotherapy, with consistent benefits even among patients aged >75 years or those with high comorbidity burden (65). These findings highlight the importance of integrating real-world evidence into clinical decision-making.

For frail patients who are ineligible for standard chemotherapy, chemotherapy-free regimens combining BV and PD-1 inhibitors have shown encouraging activity, achieving high response rates and durable disease control in small prospective studies (66).

A major advance in this population is the development of frailty-based risk stratification models. A simplified scoring system incorporating age ≥70 years, ECOG performance status ≥2, and comorbidity indices (e.g. CIRS-G or CCI) enables classification into fit, unfit, and frail subgroups (62, 67). These categories demonstrate markedly different survival outcomes and can guide treatment intensity decisions.

Overall, the management of elderly HL has transitioned toward precision stratification. The integration of novel agents, functional assessment tools, and real-world evidence enables more individualised treatment approaches. By aligning therapeutic intensity with patient fitness and biological risk, this strategy represents a critical step toward extending the concept of “functional cure” to a historically underserved population.

## 4 Evolution of treatment strategies in relapsed/refractory Hodgkin lymphoma

Historically, with conventional chemotherapy, approximately 5%–10% of patients exhibited primary refractory disease and 10%–30% relapsed after initial remission (1). For patients with relapsed/refractory (R/R) HL, the therapeutic objective is to achieve deep remission in order to enable potentially curative consolidation strategies. In the era of targeted and immunotherapy, the treatment paradigm has shifted dramatically—from cytotoxic chemotherapy–based approaches toward biologically driven, less toxic, and more effective strategies.

### 4.1 “Chemotherapy-light” salvage strategies

Traditional salvage regimens, such as ICE, DHAP, and GVD, have historically been the backbone of second-line therapy. However, these approaches are associated with substantial toxicity, require hospitalisation, and may impair stem cell mobilisation (68, 69). In contrast, regimens incorporating BV and PD-1 inhibitors have ushered in a “chemotherapy-light” paradigm (70, 71).

Real-world evidence has highlighted the limitations of conventional chemotherapy in the modern era (72). In patients relapsing after intensified frontline regimens such as eBEACOPDac, traditional second-line chemotherapy achieved poor outcomes, with a 2-year PFS of only ~32%, significantly inferior to historical controls (73). By contrast, targeted and immunotherapy-based approaches—including BV-containing regimens and PD-1–based combinations—achieved markedly superior outcomes, with 2-year PFS approaching 80% and higher rates of successful transition to autologous stem cell transplantation (ASCT) (73).

The phase III KEYNOTE−204 trial established PD−1 blockade as superior to BV monotherapy in R/R HL. Pembrolizumab significantly improved median PFS compared with BV (13.2 vs 8.3 months), with consistent benefit across treatment lines (72). Subgroup analyses further confirmed that pembrolizumab was superior to BV regardless of prior lines of therapy: among patients who received only 1 prior line, median PFS was 16.4 months vs 8.4 months; among those who received ≥2 prior lines, median PFS was 12.6 months vs 8.2 months (74).

Among combination strategies, the pembrolizumab plus GVD regimen represents a major breakthrough (75). In a phase II study, this combination achieved an unprecedented ORR of 100% and CR rate of 95%, with the vast majority of patients successfully bridged to ASCT. Long-term follow-up demonstrated durable disease control, with 5-year PFS and overall survival (OS) exceeding 90% (76).

The cross-age cooperative model has also been prospectively validated. The phase II CheckMate 744 trial, led by the Children’s Oncology Group but enrolling patients up to 30 years of age, evaluated a transplant-free, risk-adapted regimen of nivolumab plus BV in children, adolescents, and young adults (CAYA) with low-risk relapsed cHL, achieving a 3-year PFS of 95% without routine ASCT (77). In the same trial, standard-risk patients achieved a 1-year PFS of 91% (78). Additional studies in the CAYA population have confirmed the feasibility of transplant-free approaches, including the BVB regimen (brentuximab vedotin plus bendamustine) with a CR rate of 66% (79).

In the adult population, BV-based and PD-1-based strategies have similarly challenged the ASCT-mandatory paradigm. LaCasce et al. demonstrated that BV plus bendamustine as first salvage therapy achieved a CR rate of 73.6% and estimated 2-year PFS of 62.6% (80). The nivolumab-NICE regimen in high-risk adult R/R cHL achieved an ORR of 100%, CR rate of 86%, and 2-year PFS of 88% (81). Of historical relevance, single-agent BV induced durable responses in patients relapsing after ASCT, with a 5-year OS of 41% in the overall population and a 5-year OS of 64% in those achieving CR (82).

Taken together, salvage therapy in R/R HL has undergone a fundamental transformation. PD-1–based regimens now represent the backbone of treatment—either as monotherapy or in combination—while conventional chemotherapy increasingly assumes a secondary role. This shift reflects not only improved efficacy but also reduced toxicity, aligning closely with the principles of functional cure. (**Table 2**: Relapsed/Refractory HL – Chemotherapy-Light & Immunotherapy Approaches).

**Table 2:** Relapsed/refractory HL – chemotherapy-light & immunotherapy approaches.

Trial/Study	Patient population	Age/Stage	Regimen	Phase	Follow-up duration	Key endpoint(s)	Outcome	Transplant (Yes/No/Optional)	Reference
KEYNOTE-204	R/R HL	Adults	Pembrolizumab vs Brentuximab vedotin	III	Median 25.7 months (IQR 23.4–33.0)	PFS, objective response rate, safety	Median PFS 13.2 months (pembro) vs 8.3 months (BV); HR 0.65; better safety profile with pembrolizumab	Optional (some patients received subsequent autologous or allogeneic HSCT)	(72, 74)
Pembrolizumab-GVD (MSK/UM study)	R/R HL,transplant-eligible	Adults	Pembrolizumab + GVD	II	Median 57 months	ORR, CR, PFS	CR 95%; ORR 100%; 5-year PFS 91%; 5-year OS 94%; manageable safety profile	Yes (bridged to ASCT)	(75, 76)
CheckMate 744 (low-risk)	R/R cHL	Children & adolescents 5–30 years	Nivolumab + BV ×4 cycles; suboptimal responders: BV + Bendamustine ×2 cycles; CMR → ISRT 30 Gy	II	Median 31.9 months (range 2.2–55.3)	CMR any time before ISRT (primary), 3-year EFS, ORR, PFS	CMR 93% any time before ISRT; ORR 100%; 3-year EFS 87%; 3-year PFS 95%; safety profile manageable	No	(77)
CheckMate 744 (standard-risk)	R/R cHL	Children & adolescents 5–30 years	Nivolumab + BV induction ×4 cycles; suboptimal responders: BV + Bendamustine ×2 cycles; CMR → auto-HCT consolidation	II	Minimum follow-up 15.6 months	CMR any time before consolidation (primary), ORR, PFS	CMR 59% after induction; 94% before consolidation; 1-yr PFS 91%	Optional (auto‑HCT)	(78)
BVB (Forlenza)	R/R cHL	Pediatric/Adolescent	BV + Bendamustine	Retrospective, multicenter	Median 36 months	CMR (primary), ORR, 3-year EFS, 3-year OS	CMR 66%; ORR 79%; 3-year EFS 65%; 3-year OS 89%; manageable toxicity	Yes (16 patients: 13 autologous, 3 allogeneic)	(79)
BVB (LaCasce)	R/R cHL	Adults	BV + Bendamustine;optional post-ASCT BV monotherapy	I/II	Median 23.0 months (range 4–34)	CR (primary), ORR, PFS, OS	CR 73.6% (39/53); ORR 92.5%; 2-year PFS 62.6% overall, 69.8% in those receiving ASCT; 2-year OS 94.2% overall, 94.9% in ASCT patients	Yes/Optional (40 patients underwent ASCT)	(80)
Nivolumab-NICE (Mei)	R/R HL	Adults	Nivolumab + ICE	II	Median 31.2 months (range 7.8–55)	CR (primary), ORR, 2‑year PFS, 2‑year OS	ORR 100%; CR 86%; 2-year PFS 88%; 2-year OS 100%; post-ASCT 2-year PFS 94%, OS 100%; manageable toxicity	Yes/Optional (32/35 proceeded to ASCT; others per physician discretion)	(81)
BV – pivotal phase II	R/R HL after failed autologous SCT	Adults	Single-agent BV	II	Median 35.1 months (range 1.8–72.9) from first dose	CR (primary), ORR, PFS, OS	CR (33%); ORR 75%; 5-year PFS 22% overall, 52% in CR subset; 5-year OS 41% overall, 64% in CR subset; manageable toxicity	Optional (22 patients underwent SCT; 8 allo-SCT; 9 CR patients remain in remission without further therapy)	(82)

### 4.2 Overcoming resistance to PD-1 blockade

With the widespread use of PD-1 inhibitors, acquired resistance has emerged as a major clinical challenge (83). Resistance mechanisms are multifactorial, involving tumour-intrinsic genetic alterations, dysregulated signalling pathways,and an immunosuppressive microenvironment. From a biological perspective, 9p24.1 amplification and PD-L1 overexpression underpin initial sensitivity to PD-1 blockade (32), whereas loss of MHC class II expression has been associated with poor response (84). Persistent activation of the JAK–STAT pathway further contributes to resistance by modulating interferon signalling and immune escape (85, 86). Additionally, epigenetic reprogramming and the accumulation of immunosuppressive cell populations within the tumour microenvironment play critical roles (87).

Epigenetic therapy combined with PD-1 blockade has emerged as one of the most promising approaches to overcoming resistance. The combination of decitabine and camrelizumab (DP regimen) significantly improved outcomes compared with PD-1 monotherapy, achieving higher complete response rates (79% vs 32%) and prolonged PFS (35.0 vs 15.5 months) (88). Mechanistically, hypomethylating agents restore immune sensitivity by enhancing antigen presentation and reversing T-cell exhaustion (88, 89). Importantly, this strategy retains efficacy in PD-1–pretreated patients, with response rates exceeding 50% and meaningful prolongation of PFS (90). Building on this, triplet regimens incorporating histone deacetylase inhibitors (e.g. chidamide) have demonstrated even greater activity. The CDP regimen achieved an ORR of 94% and CR rate of 50% in heavily pretreated patients, including those resistant to prior epigenetic therapy (87). Single-cell analyses revealed that this approach reprogrammes the tumour microenvironment by enhancing cytotoxic CD8^+^ T-cell activity and suppressing immunosuppressive T-cell subsets (87).

Given its central role in immune escape, the JAK–STAT pathway represents another rational therapeutic target. The combination of ruxolitinib and nivolumab demonstrated encouraging activity in PD-1–refractory patients, with response rates exceeding 50% (85). Mechanistically, JAK inhibition reduces myeloid-derived suppressor cells and restores T-cell function, thereby enhancing the efficacy of immune checkpoint blockade (85).

Interestingly, chemotherapy rechallenge remains a viable option after PD-1 failure. Retrospective studies have shown high response rates (>90%) even in patients previously refractory to chemotherapy (91), suggesting that PD-1 blockade may re-sensitise tumour cells to cytotoxic agents. In resource-limited settings, cost-effective regimens such as BeGEV have demonstrated high response rates and favourable safety profiles, providing a practical alternative where access to novel agents is restricted (92).

In summary, PD-1 resistance reflects a complex interplay between tumour genetics, signalling pathways, and immune microenvironment. Emerging strategies—including epigenetic modulation, JAK inhibition, and rational combination therapies—are beginning to overcome this barrier. Future research must focus on biomarker-driven selection of these approaches to optimise patient outcomes.

### 4.3 Emerging targeted and cellular therapies

For patients with dual resistance to BV and PD-1 inhibitors, therapeutic options remain extremely limited. This has driven the development of next-generation targeted agents and cellular therapies.

PF-08046044 (35C) is a novel CD30-directed antibody–drug conjugate delivering a topoisomerase I inhibitor payload (93). In early-phase studies involving heavily pretreated patients (median ≥6 prior lines), this agent achieved an ORR of 76% (93). Notably, ctDNA analyses demonstrated rapid and profound molecular responses, suggesting a strong correlation between early molecular clearance and clinical efficacy (93).

AFM13, a CD30/CD16A bispecific antibody, redirects natural killer (NK) cells to target HRS cells (94, 95). While monotherapy activity is modest (96, 97), combination strategies have yielded remarkable results.In a phase I study combining AFM13 with cord blood–derived, cytokine-activated NK cells, the ORR reached 97.3%, with a CR rate of 73% in heavily pretreated patients (94). Importantly, this “off-the-shelf” cellular therapy demonstrated a favourable safety profile without significant cytokine release syndrome or neurotoxicity (94).

MT-601 represents a novel autologous T-cell product targeting multiple tumour-associated antigens (e.g. PRAME, NY-ESO-1, MAGE-A4) (98). In early clinical evaluation, this approach achieved an ORR of 78% in patients who had failed BV, PD-1 inhibitors, and transplantation (98). This multi-target strategy may help overcome tumour heterogeneity and antigen escape.

Collectively, these emerging therapies highlight a shift toward highly personalised, immune-based treatment strategies. While still in early development, they offer promising options for patients with otherwise limited therapeutic alternatives and represent the next frontier in HL management.

## 5 Re-evaluating the role of transplantation in the modern era

High-dose chemotherapy followed by autologous stem cell transplantation (HDT-ASCT) has served as the cornerstone of consolidation therapy for relapsed/refractory HL for over three decades (99). However, in the era of highly effective targeted and immunotherapeutic agents, the role of transplantation is undergoing profound reassessment (100–102).

### 5.1 The enduring role of autologous transplantation

Traditionally, ASCT has achieved cure rates of approximately 50%–60% following salvage chemotherapy (102). The introduction of BV and PD-1 inhibitors into salvage regimens has significantly improved pre-transplant response rates, with overall response rates exceeding 85% and complete response rates reaching up to 86% (81, 103, 104). These improvements have translated into better post-transplant outcomes, reinforcing the continued relevance of ASCT as a consolidation strategy.

For patients at high risk of post-transplant relapse, consolidation therapy with BV has demonstrated clear benefit (104). The AETHERA trial showed that BV maintenance significantly improved progression-free survival compared with placebo, with durable benefit observed in long-term follow-up (105). Meta-analytic data further support this strategy, demonstrating sustained improvements in both progression-free and overall survival across real-world populations (104). More recently, a multicenter phase II trial evaluated the combination of BV plus nivolumab as post-ASCT consolidation in high-risk patients with relapsed/refractory HL, reporting an 18−month progression−free survival of 94% (106).

### 5.2 Emerging concept: transplant omission

A central question in the modern era is whether all patients achieving deep remission with novel agents require transplantation. Real-world data suggest that this may not be the case (107). In patients achieving complete response following salvage therapy, outcomes with non-transplant approaches appear comparable to those undergoing ASCT, particularly when remission is achieved with PD-1– or BV-based regimens (107).

These findings challenge the historical paradigm in which ASCT was considered mandatory for all transplant-eligible patients. Instead, they support a more individualised approach, in which the depth and biology of response—rather than eligibility alone—guide consolidation decisions.

### 5.3 Role of allogeneic transplantation

For patients who relapse after ASCT or have primary refractory disease, allogeneic stem cell transplantation (allo-HSCT) remains the only potentially curative option (108). Advances in conditioning regimens, supportive care, and graft-versus-host disease (GVHD) prophylaxis have improved outcomes and reduced treatment-related mortality (109). Importantly, prior exposure to PD-1 inhibitors introduces both opportunities and challenges. While it reduces relapse risk, it may increase the incidence of acute GVHD (108). The use of post-transplant cyclophosphamide (PTCy) has been shown to mitigate this risk while preserving survival benefits (108).

To further reduce transplant−related toxicity, reduced−intensity conditioning (RIC) has emerged as a preferred approach in allo−HSCT for Hodgkin lymphoma, as it significantly lowers non−relapse mortality (NRM) while preserving graft−versus−lymphoma effects. In the prospective HDR−ALLO study, RIC allo−HSCT was associated with a non−relapse mortality rate of approximately 8% at 100 days and 15% at 1 year, and the PFS rate for allografted patients was about 48% at 1 year and 24% at 4 years in heavily pretreated relapsed/refractory HL patients (110). Subsequent registry analyses and single−center series have further supported the feasibility, acceptable NRM, and long−term disease control offered by RIC−based allo−HSCT in this setting.

A separate study directly comparing checkpoint inhibitors (CPI) versus BV as pre-transplant salvage therapy further supported the benefit of CPI, demonstrating a 5-year post-transplant relapse rate of only 5% in the CPI group compared with 37% in the BV group (p = 0.002), with CPI identified as the sole independent predictor of relapse (HR 0.124) (111). These findings highlight the importance for careful integration of immunotherapy into transplant pathway.

Overall, the role of transplantation in HL is transitioning from a universal standard to a more individualized and context-dependent strategy. ASCT remains important, but its indication is increasingly refined according to treatment response, biological risk, and prior exposure to novel agents. This evolution reflects a broader shift toward personalised, response-adapted care aligned with the goals of functional cure.

## 6 Integration of precision medicine in Hodgkin lymphoma

Advances in precision medicine are transforming HL management from traditional clinical staging toward multidimensional, biomarker-driven strategies. The integration of prognostic models, ctDNA, and serum biomarkers is enabling more accurate risk stratification and dynamic treatment adaptation.

### 6.1 Evolution of prognostic models: From IPS to A-HIPI/E-HIPI

The International Prognostic Score (IPS), developed in 1998, has long served as the standard tool for risk stratification in advanced HL (112). However, its predictive value has diminished in the context of modern therapies (112).

Newer models, such as the Advanced-stage Hodgkin Lymphoma International Prognostic Index (A-HIPI), have been developed using contemporary datasets. A-HIPI incorporates key clinical variables—including age, stage, tumour bulk, and laboratory parameters—and demonstrates robust predictive value independent of treatment modality (113). Importantly, recent analyses indicate that A-HIPI retains prognostic relevance throughout the disease course, influencing outcomes even after initial remission (114).

Newer models, such as the Early-stage HL International Prognostic Index (E-HIPI), have been developed for early-stage disease. E-HIPI incorporates key clinical variables—including sex, maximum tumour diameter, albumin, and haemoglobin—and demonstrates robust predictive value across different treatment settings (115). Importantly, external validation in patients receiving intensive chemotherapy (e.g. BEACOPP-based regimens) confirmed its performance as a treatment-agnostic tool, with a C-statistic of 0.65 and good calibration (116). These models outperform traditional IPS and offer improved risk discrimination across treatment settings.

However, current models remain limited by their reliance on static clinical variables and lack integration of dynamic biomarkers. Future prognostic systems will likely incorporate imaging, ctDNA, and multi-omics data to enable truly personalised risk prediction.

### 6.2 Clinical utility of ctDNA monitoring

Circulating tumour DNA has emerged as one of the most promising tools in HL precision medicine, offering a minimally invasive and dynamic assessment of tumour burden and molecular characteristics.

A large meta-analysis demonstrated that ctDNA has prognostic value across all treatment stages. High baseline ctDNA levels are associated with inferior survival, while persistent ctDNA positivity during or after treatment strongly predicts relapse (117). Notably, ctDNA measured at the end of treatment represents the most powerful predictor of outcome (117).

Prospective analyses, including those derived from the SWOG S1826 trial, further highlight the superiority of ctDNA over conventional imaging (118). Early ctDNA dynamics can stratify patients into distinct risk groups, with those achieving rapid molecular clearance exhibiting outcomes comparable to ctDNA-negative patients, while those with suboptimal decline have markedly poor prognosis (118).

Integration of ctDNA with PET/CT further refines risk stratification. Patients negative for both ctDNA and PET have excellent outcomes, whereas dual-positive patients represent an ultra-high-risk group (119). A critical clinical question is the concordance and discordance between ctDNA and PET/CT. While both modalities provide complementary information, discordant cases occur and carry distinct prognostic implications. For instance, patients who achieve PET negativity but remain ctDNA−positive have been shown to harbor a significantly higher risk of subsequent relapse compared with those who are negative by both tests, suggesting that ctDNA can detect minimal residual disease not captured by metabolic imaging (43). Conversely, PET−positive but ctDNA−negative cases may represent inflammatory or post−treatment changes rather than true residual disease, potentially averting unnecessary treatment intensification. The prognostic superiority of ctDNA over interim PET has been further demonstrated in the SWOG S1826 trial, where early ctDNA clearance was more strongly associated with long−term outcome than PET response (118). These observations support the integration of both modalities into response−adapted algorithms, with ctDNA offering a dynamic, quantitative measure of molecular burden that can guide de−escalation or intensification strategies, particularly in patients with equivocal PET findings.This combined approach enables more precise identification of patients who may benefit from treatment intensification or de-escalation.

Advances in detection technologies have also improved sensitivity and clinical applicability. High-resolution platforms enable the detection of minimal residual disease (MRD), providing an opportunity to guide treatment decisions in real time.

### 6.3 Serum biomarkers and microenvironmental signatures

In addition to ctDNA, soluble biomarkers reflecting tumour–microenvironment interactions are gaining increasing attention.

TARC (CCL17) is one of the most characteristic biomarkers in HL.Produced by HRS cells, TARC plays a key role in recruiting CCR4-positive T cells and shaping the tumour microenvironment (36). Clinically, serum TARC levels correlate with tumour burden and respond rapidly to effective therapy, making it a valuable tool for monitoring treatment response (120, 121). Notably, TARC may outperform interim PET in predicting progression-free survival, particularly when assessed dynamically during treatment. Post-treatment TARC monitoring can also detect relapse earlier than clinical or radiographic progression (122, 123).

Other biomarkers, including soluble CD30 and interleukin-6, have been associated with disease risk and may reflect early tumour development and immune activation (124, 125). These markers show potential for early detection and risk prediction, although their clinical utility requires further validation.

Importantly, the integration of ctDNA and serum biomarkers represents a powerful strategy for capturing both tumour-intrinsic and microenvironmental dynamics. This multidimensional approach is likely to surpass single-modality assessment and play a central role in future precision-guided treatment.

## 7 Conclusions and future perspectives

The treatment of Hodgkin lymphoma is undergoing a profound paradigm shift. In the past, the overriding principle was “cure at all costs,” with survival being the sole measure of success. Today, building on the substantial efficacy gains achieved with novel agents such as brentuximab vedotin and immune checkpoint inhibitors (e.g., PD−1 inhibitors), we are able to pursue a more ambitious goal: functional cure(i.e., durable remission with minimal long−term toxicity, preserving organ function and quality of life). This new paradigm centres on preserving long−term disease control while maximising the protection of organ function, fertility, neurological integrity, and mental health—ensuring that patients not only live longer but also live better (126, 127). Although the individual components of this approach have been pursued for decades, the term “functional cure” unifies them into a coherent framework for modern HL management.

Despite remarkable progress, several challenges remain. First, accurately identifying patients eligible for treatment de−escalation is a critical issue. Although tools such as ctDNA have shown promise, standardised algorithms for integrating ctDNA into clinical decision−making are still lacking. Prospective studies are urgently needed to validate whether ctDNA−guided de−escalation can substantially reduce toxicity without compromising survival (128). Second, overcoming resistance to immunotherapy is a major challenge. Mechanisms of PD−1 resistance are complex, involving epigenetic regulation, the JAK–STAT pathway, the tumour microenvironment, and metabolic reprogramming, among others. Future efforts should focus on mechanism−driven combination strategies. Agents targeting novel immune checkpoints such as the CD86–CTLA−4 axis, LAG−3, and TIM−3 are entering clinical development and may provide new options for patients with resistant disease (129). Third, managing the long−term toxicities of novel agents deserves attention. The long−term immune−related adverse events of immune checkpoint inhibitors and their effects on fertility remain incompletely characterised. Although brentuximab vedotin−induced neuropathy is reversible in most cases, some patients experience persistent symptoms. Establishing long−term follow−up systems in the era of novel therapies is essential (130–132). Finally, improving global access to novel agents is a practical concern. High−cost drugs such as brentuximab vedotin and PD−1 inhibitors are not equally available worldwide, and developing effective, affordable alternatives is critical for resource−limited settings (133).

Looking ahead, the management of Hodgkin lymphoma will continue to evolve along the trajectory of “precision staging, dynamic adaptation, and life−course care.” At diagnosis, multidimensional prognostic models integrating clinical variables, tumour burden, molecular features, and genomic information will be established (134). During treatment, real−time adjustments to treatment intensity and duration will be guided by dynamic ctDNA monitoring and interim PET/CT. After treatment completion, individualised long−term surveillance plans will be based on risk stratification, with emphasis on monitoring second malignancies, cardiovascular events, endocrine function, and mental health, supported by multidisciplinary survivorship programmes (135). Ultimately, as we deepen our understanding of HL biology, continue to develop novel drugs and cellular therapies, and increasingly apply precision medicine tools, functional cure will transition from being the privilege of a few to a realistic expectation for every patient with Hodgkin lymphoma.

## References

[1] Ansell SM . Hodgkin lymphoma: 2025 update on diagnosis, risk-stratification, and management. Am J Hematol. (2024) 99:2367–78. doi: 10.1002/ajh.27470. PMID: 39239794

[2] Huang J Pang WS Lok V Zhang L Lucero-Prisno DE Xu W . Incidence, mortality, risk factors, and trends for Hodgkin lymphoma: a global data analysis. J Hematol Oncol. (2022) 15:57. doi: 10.1186/s13045-022-01281-9. PMID: 35546241 PMC9097358

[3] Siegel RL Kratzer TB Giaquinto AN Sung H Jemal A . Cancer statistics, 2025. CA Cancer J Clin. (2025) 75:10–45. doi: 10.3322/caac.21871. PMID: 39817679 PMC11745215

[4] Sun K Wu H Zhu Q Gu K Wei H Wang S . Global landscape and trends in lifetime risks of haematologic Malignancies in 185 countries: population-based estimates from Globocan 2022. EClinicalMedicine. (2025) 83:103193. doi: 10.1016/j.eclinm.2025.103193. PMID: 40256772 PMC12008131

[5] Shao K Wang C Chen W Gong W Lin X Ye B . Burden of hematologic Malignancies in China from 1990 to 2021: analysis from the Global Burden of Disease Study 2021. BMC Public Health. (2025) 25:2280. doi: 10.1186/s12889-025-23469-7. PMID: 40604781 PMC12220249

[6] Brice P de Kerviler E Friedberg JW . Classical hodgkin lymphoma. Lancet. (2021) 398:1518–27. doi: 10.1016/s0140-6736(20)32207-8. PMID: 33493434

[7] Lu Z Teng Y Ning X Wang H Feng W Ou C . Long-term risk of cardiovascular disease mortality among classic Hodgkin lymphoma survivors. Cancer. (2022) 128:3330–9. doi: 10.1002/cncr.34375. PMID: 35872619

[8] Barrett A Shah N Chadwick A Burns D Burton C Cutter DJ . Assessment of fitness for bleomycin use and management of bleomycin pulmonary toxicity in patients with classical Hodgkin lymphoma: a British Society for Haematology good practice paper. Br J Haematol. (2025) 206:74–85. doi: 10.1111/bjh.19840. PMID: 39506502

[9] Drechsel KCE Pilon MCF Stoutjesdijk F Meivis S Schoonmade LJ Wallace WHB . Reproductive ability in survivors of childhood, adolescent, and young adult Hodgkin lymphoma: a review. Hum Reprod Update. (2023) 29:486–517. doi: 10.1093/humupd/dmad002. PMID: 36779325 PMC10320502

[10] Herrera AF LeBlanc M Castellino SM Li H Rutherford SC Evens AM . Nivolumab+AVD in advanced-stage classic Hodgkin's lymphoma. N Engl J Med. (2024) 391:1379–89. doi: 10.1056/NEJMoa2405888. PMID: 39413375 PMC11488644

[11] Ferdinandus J Schneider G Moccia A Greil R Hertzberg M Schaub V . Fertility in patients with advanced-stage classic Hodgkin lymphoma treated with BRECADD versus EBEACOPP: a secondary analysis of the multicentre, randomised, parallel, open-label, phase 3 HD21 trial. Lancet Oncol. (2025) 26:1081–90. doi: 10.1016/s1470-2045(25)00262-1. PMID: 40652943

[12] Alibrahim MN Gloghini A Carbone A . Classic Hodgkin lymphoma: pathobiological features that impact emerging therapies. Blood Rev. (2025) 71:101271. doi: 10.1016/j.blre.2025.101271. PMID: 39904647

[13] Piris MA Medeiros LJ Chang KC . Hodgkin lymphoma: a review of pathological features and recent advances in pathogenesis. Pathology. (2020) 52:154–65. doi: 10.1016/j.pathol.2019.09.005. PMID: 31699300

[14] Weniger MA Küppers R . Molecular biology of Hodgkin lymphoma. Leukemia. (2021) 35:968–81. doi: 10.1038/s41375-021-01204-6. PMID: 33686198 PMC8024192

[15] Kosydar S Ansell SM . The biology of classical Hodgkin lymphoma. Semin Hematol. (2024) 61:212–20. doi: 10.1053/j.seminhematol.2024.05.001. PMID: 38824068

[16] van der Weyden CA Pileri SA Feldman AL Whisstock J Prince HM . Understanding CD30 biology and therapeutic targeting: a historical perspective providing insight into future directions. Blood Cancer J. (2017) 7:e603. doi: 10.1038/bcj.2017.85. PMID: 28885612 PMC5709754

[17] Wang Y Nowakowski GS Wang ML Ansell SM . Advances in CD30- and PD-1-targeted therapies for classical Hodgkin lymphoma. J Hematol Oncol. (2018) 11:57. doi: 10.1186/s13045-018-0601-9. PMID: 29685160 PMC5914042

[18] Matsuki E Younes A . Lymphomagenesis in Hodgkin lymphoma. Semin Cancer Biol. (2015) 34:14–21. doi: 10.1016/j.semcancer.2015.02.002. PMID: 25725205

[19] Küppers R . Advances in Hodgkin lymphoma research. Trends Mol Med. (2025) 31:326–43. doi: 10.1016/j.molmed.2024.10.004. PMID: 39443214

[20] Weniger MA Küppers R . NF-κB deregulation in Hodgkin lymphoma. Semin Cancer Biol. (2016) 39:32–9. doi: 10.1016/j.semcancer.2016.05.001. PMID: 27221964

[21] Hinz M Löser P Mathas S Krappmann D Dörken B Scheidereit C . Constitutive NF-kappaB maintains high expression of a characteristic gene network, including CD40, CD86, and a set of antiapoptotic genes in Hodgkin/Reed-Sternberg cells. Blood. (2001) 97:2798–807. doi: 10.1182/blood.v97.9.2798. PMID: 11313274

[22] Tiacci E Ladewig E Schiavoni G Penson A Fortini E Pettirossi V . Pervasive mutations of JAK-STAT pathway genes in classical Hodgkin lymphoma. Blood. (2018) 131:2454–65. doi: 10.1182/blood-2017-11-814913. PMID: 29650799 PMC6634958

[23] Ok CY Young KH . Checkpoint inhibitors in hematological Malignancies. J Hematol Oncol. (2017) 10:103. doi: 10.1186/s13045-017-0474-3. PMID: 28482851 PMC5422942

[24] Dutton A Reynolds GM Dawson CW Young LS Murray PG . Constitutive activation of phosphatidyl-inositide 3 kinase contributes to the survival of Hodgkin’s lymphoma cells through a mechanism involving Akt kinase and mTOR. J Pathol. (2005) 205:498–506. doi: 10.1002/path.1725. PMID: 15714459

[25] Georgakis GV Li Y Rassidakis GZ Medeiros LJ Mills GB Younes A . Inhibition of the phosphatidylinositol-3 kinase/Akt promotes G1 cell cycle arrest and apoptosis in Hodgkin lymphoma. Br J Haematol. (2006) 132:503–11. doi: 10.1111/j.1365-2141.2005.05881.x. PMID: 16412023

[26] Vrzalikova K Ibrahim M Vockerodt M Perry T Margielewska S Lupino L . S1PR1 drives a feedforward signalling loop to regulate BATF3 and the transcriptional programme of Hodgkin lymphoma cells. Leukemia. (2018) 32:214–23. doi: 10.1038/leu.2017.275. PMID: 28878352 PMC5737877

[27] Xie L Ushmorov A Leithäuser F Guan H Steidl C Färbinger J . FOXO1 is a tumor suppressor in classical Hodgkin lymphoma. Blood. (2012) 119:3503–11. doi: 10.1182/blood-2011-09-381905. PMID: 22343918

[28] Georgoulis V Papoudou-Bai A Makis A Kanavaros P Hatzimichael E . Unraveling the immune microenvironment in classic Hodgkin lymphoma: prognostic and therapeutic implications. Biol (Basel). (2023) 12(6):862. doi: 10.3390/biology12060862. PMID: 37372147 PMC10294989

[29] Aldinucci D Celegato M Casagrande N . Microenvironmental interactions in classical Hodgkin lymphoma and their role in promoting tumor growth, immune escape and drug resistance. Cancer Lett. (2016) 380:243–52. doi: 10.1016/j.canlet.2015.10.007. PMID: 26474544

[30] Xie W Medeiros LJ Li S Yin CC Khoury JD Xu J . PD-1/PD-L1 pathway and its blockade in patients with classic Hodgkin lymphoma and non-Hodgkin large-cell lymphomas. Curr Hematol Malig Rep. (2020) 15:372–81. doi: 10.1007/s11899-020-00589-y. PMID: 32394185

[31] García-Montenegro M Narbaitz M Metrebian MF Pavlovsky A Slavutsky I . PD-L1/PD-L2 genetic profile in the molecular cytogenetic classification of classic Hodgkin lymphoma. Virchows Arch. (2025) 486:1039–47. doi: 10.1007/s00428-025-04047-z. PMID: 39976683

[32] Liu WR Shipp MA . Signaling pathways and immune evasion mechanisms in classical Hodgkin lymphoma. Blood. (2017) 130:2265–70. doi: 10.1182/blood-2017-06-781989. PMID: 29167175 PMC5701523

[33] Carbone A Gloghini A Carlo-Stella C . Are EBV-related and EBV-unrelated Hodgkin lymphomas different with regard to susceptibility to checkpoint blockade? Blood. (2018) 132:17–22. doi: 10.1182/blood-2018-02-833806. PMID: 29716887

[34] Wienand K Chapuy B Stewart C Dunford AJ Wu D Kim J . Genomic analyses of flow-sorted Hodgkin Reed-Sternberg cells reveal complementary mechanisms of immune evasion. Blood Adv. (2019) 3:4065–80. doi: 10.1182/bloodadvances.2019001012. PMID: 31816062 PMC6963251

[35] Kilsdonk M Veldman C Rosati S Plattel W Diepstra A . The value of thymus and activation related chemokine immunohistochemistry in classic Hodgkin lymphoma diagnostics. Histopathology. (2023) 82:495–503. doi: 10.1111/his.14836. PMID: 36345263 PMC10100154

[36] Zijtregtop EAM van der Strate I Beishuizen A Zwaan CM Scheijde-Vermeulen MA Brandsma AM . Biology and clinical applicability of plasma thymus and activation-regulated chemokine (TARC) in classical Hodgkin lymphoma. Cancers (Basel). (2021) 13(4):884. doi: 10.3390/cancers13040884. PMID: 33672548 PMC7923750

[37] Plattel WJ van den Berg A Visser L van der Graaf AM Pruim J Vos H . Plasma thymus and activation-regulated chemokine as an early response marker in classical Hodgkin's lymphoma. Haematologica. (2012) 97:410–15. doi: 10.3324/haematol.2011.053199. PMID: 22058214 PMC3291596

[38] Patel SS Weirather JL Lipschitz M Lako A Chen PH Griffin GK . The microenvironmental niche in classic Hodgkin lymphoma is enriched for CTLA-4-positive T cells that are PD-1-negative. Blood. (2019) 134:2059–69. doi: 10.1182/blood.2019002206. PMID: 31697809 PMC7218752

[39] Gottschlich A Grünmeier R Hoffmann GV Nandi S Kavaka V Müller PJ . Dissection of single-cell landscapes for the development of chimeric antigen receptor T cells in Hodgkin lymphoma. Blood. (2025) 145:1536–52. doi: 10.1182/blood.2023022197. PMID: 40178843 PMC12002222

[40] Lim S Jeon YK . Liquid biopsies for Hodgkin lymphoma. Nat Rev Cancer. (2024) 24:522. doi: 10.1038/s41568-024-00709-3. PMID: 38783096

[41] Wang H Wang Z Zhu S Li Z Yang H Sun P . Circulating tumor DNA assisting lymphoma genetic feature profiling and identification. Ann Hematol. (2024) 103:4135–44. doi: 10.1007/s00277-024-05782-0. PMID: 39012515

[42] Pirosa MC Bruscaggin A Terzi di Bergamo L Salehi M Jauk F Forestieri G . A comprehensive genetic study of classic Hodgkin lymphoma using circulating tumor DNA. Blood. (2025) 146:1207–24. doi: 10.1182/blood.2024027355. PMID: 40359477

[43] Heger JM Mammadova L Mattlener J Sobesky S Cirillo M Altmüller J . Circulating tumor DNA sequencing for biologic classification and individualized risk stratification in patients with Hodgkin lymphoma. J Clin Oncol. (2024) 42:4218–30. doi: 10.1200/jco.23.01867. PMID: 39348625

[44] Lagerlöf I Fohlin H Enblad G Glimelius B Goldkuhl C Palma M . Limited, but not eliminated, excess long-term morbidity in stage I-IIA Hodgkin lymphoma treated with doxorubicin, bleomycin, vinblastine, and dacarbazine and limited-field radiotherapy. J Clin Oncol. (2022) 40:1487–96. doi: 10.1200/jco.21.02407. PMID: 35077204 PMC9061145

[45] van Leeuwen FE Ng AK . Long-term risk of second Malignancy and cardiovascular disease after Hodgkin lymphoma treatment. Hematol Am Soc Hematol Educ Program. (2016) 2016:323–30. doi: 10.1182/asheducation-2016.1.323. PMID: 27913498 PMC6142518

[46] Radford J Illidge T Counsell N Hancock B Pettengell R Johnson P . Results of a trial of PET-directed therapy for early-stage Hodgkin’s lymphoma. N Engl J Med. (2015) 372:1598–607. doi: 10.1056/NEJMoa1408648. PMID: 25901426

[47] André MPE Girinsky T Federico M Reman O Fortpied C Gotti M . Early positron emission tomography response-adapted treatment in stage I and II Hodgkin lymphoma: final results of the randomized EORTC/LYSA/FIL H10 trial. J Clin Oncol. (2017) 35:1786–94. doi: 10.1200/jco.2016.68.6394. PMID: 28291393

[48] Radford J Williams J Edwards D Pettengell R Johnson P Hoskin P . Involved field radiotherapy versus no further treatment in patients with newly diagnosed stage 1A or 2A Hodgkin lymphoma and a ‘negative’ PET scan after 3 cycles ABVD. Survival and cause of death after a median of 16 years follow-up in the UK RAPID trial. Blood. (2024) 144:457. doi: 10.1182/blood-2024-200662 38484137

[49] Federico M Fortpied C Stepanishyna Y Gotti M van der Maazen R Cristinelli C . Long-term follow-up of the response-adapted intergroup EORTC/LYSA/FIL H10 trial for localized Hodgkin lymphoma. J Clin Oncol. (2024) 42:19–25. doi: 10.1200/jco.23.01745. PMID: 37967311 PMC10730029

[50] Fiaccadori V Neven A Fortpied C Aurer I Andre M Federico M . Relapse patterns in early-PET negative, limited-stage Hodgkin lymphoma (HL) after ABVD with or without radiotherapy-a joint analysis of EORTC/LYSA/FIL H10 and NCRI RAPID trials. Br J Haematol. (2023) 200:731–9. doi: 10.1111/bjh.18594. PMID: 36541117

[51] Bröckelmann PJ Bühnen I Meissner J Trautmann-Grill K Herhaus P Halbsguth TV . Nivolumab and doxorubicin, vinblastine, and dacarbazine in early-stage unfavorable Hodgkin lymphoma: Final analysis of the randomized German Hodgkin Study Group phase II Nivahl trial. J Clin Oncol. (2023) 41:1193–9. doi: 10.1200/jco.22.02355. PMID: 36508302

[52] Plattel WJ Teesink S Visser L Voltin CA Kaul H Schlößer HA . Serum TARC dynamics during anti-PD1-based first-line Hodgkin lymphoma treatment: An analysis from the GHSG Nivahl trial. Hemasphere. (2025) 9:e70205. doi: 10.1002/hem3.70205. PMID: 40862220 PMC12371470

[53] Henderson TO Hu B Keller F Pei Q Wu Y Hoppe B . Ahod2131: A randomized phase 3 response-adapted trial comparing standard therapy with immuno-oncology therapy for children and adults with newly diagnosed stage I and II classic Hodgkin lymphoma. Blood. (2023) 142:3084. doi: 10.1182/blood-2023-189652

[54] Fornecker LM Lazarovici J Aurer I Casasnovas RO Gac AC Bonnet C . Brentuximab vedotin plus AVD for first-line treatment of early-stage unfavorable Hodgkin lymphoma (BREACH): A multicenter, open-label, randomized, phase II trial. J Clin Oncol. (2023) 41:327–35. doi: 10.1200/jco.21.01281. PMID: 35867960

[55] Herrera A Leblanc M Castellino S Li H Rutherford S Evens A . 3-year follow-up of the S1826 study confirms improved progression-free survival with nivolumab-AVD compared to brentuximab vedotin-AVD in advanced stage classic Hodgkin lymphoma. Blood. (2025) 146:151. doi: 10.1182/blood-2025-151

[56] Castellino SM Li H Herrera AF LeBlanc M Parsons SK Unger JM . Three-year follow-up of nivolumab-AVD versus brentuximab vedotin-AVD in adolescents with advanced-stage classic Hodgkin lymphoma on S1826. J Clin Oncol. (2026) 44:449–54. doi: 10.1200/jco-25-00203. PMID: 41512237 PMC13192332

[57] Ferdinandus J Kaul H Moccia A Greil R Hertzberg M Schaub V . Final analysis of the phase III GHSG HD21 trial: PET-guided BRECADD vs. eBEACOPP in advanced-stage classical Hodgkin lymphoma. Blood. (2025) 146:152. doi: 10.1182/blood-2025-152

[58] Borchmann P Ferdinandus J Schneider G Moccia A Greil R Hertzberg M . Assessing the efficacy and tolerability of PET-guided BRECADD versus eBEACOPP in advanced-stage, classical Hodgkin lymphoma (HD21): A randomised, multicentre, parallel, open-label, phase 3 trial. Lancet. (2024) 404:341–52. doi: 10.1016/s0140-6736(24)01315-1. PMID: 38971175

[59] Ferdinandus J Kaul H Fosså A Hüttmann A Keil F Ko YD . Positron emission tomography-guided brentuximab vedotin, etoposide, cyclophosphamide, doxorubicin, dacarbazine, and dexamethasone in older patients with advanced-stage classic Hodgkin lymphoma: A prospective, multicenter, single-arm, phase II cohort of the German Hodgkin Study Group HD21 trial. J Clin Oncol. (2025) 43:2974–85. doi: 10.1200/jco-25-00439. PMID: 40674676

[60] Ansell SM Radford J Connors JM Długosz-Danecka M Kim WS Gallamini A . Overall survival with brentuximab vedotin in stage III or IV Hodgkin's lymphoma. N Engl J Med. (2022) 387:310–20. doi: 10.1056/NEJMoa2206125. PMID: 35830649

[61] McKenna M Ryu Tiger YK Rutherford SC Evens AM . The management of older patients with Hodgkin lymphoma: Implications of S1826. Semin Hematol. (2024) 61:236–44. doi: 10.1053/j.seminhematol.2024.05.004. PMID: 38945791

[62] Lia K Jørgensen RRK Wikman P Wold BL Övergaard N Fluge Ø . A simplified frailty score predicts outcome in curatively treated older patients with classical Hodgkin lymphoma. Haematologica. (2025) 110:2356–66. doi: 10.3324/haematol.2025.287509. PMID: 40270216 PMC12485341

[63] Bröckelmann PJ . Treatment approaches for older Hodgkin lymphoma patients. Curr Opin Oncol. (2024) 36:353–9. doi: 10.1097/cco.0000000000001071. PMID: 39005230

[64] Rutherford SC Li H Herrera AF LeBlanc M Ahmed S Davison K . Nivolumab-AVD versus brentuximab vedotin-AVD in older patients with advanced-stage classic Hodgkin lymphoma enrolled on S1826. J Clin Oncol. (2025) 43:2968–73. doi: 10.1200/jco-25-00204. PMID: 40523203 PMC12353409

[65] Peiqi Z Bai M Huang C Wang J Li Y Jiang Y . Frontline brentuximab vedotin and PD-1 inhibitors in elderly Chinese Hodgkin lymphoma: Real-world survival benefits validated by geriatric and biomarker correlates. Blood. (2025) 146:1843. doi: 10.1182/blood-2025-1843

[66] Friedberg JW Bordoni R Patel-Donnelly D Larson T Goldschmidt J Boccia R . Brentuximab vedotin with dacarbazine or nivolumab as frontline CHL therapy for older patients ineligible for chemotherapy. Blood. (2024) 143:786–95. doi: 10.1182/blood.2022019536. PMID: 37946283

[67] Moskowitz AJ . Fit, unfit, or frail? Now easier to tell for Hodgkin lymphoma. Haematologica. (2025) 110:2250–2. doi: 10.3324/haematol.2025.288033. PMID: 40671506 PMC12485319

[68] Mahdy A Hamoda A Zaher A Khorshed E Elwakeel M Hassanein O . Outcome and toxicity of ifosfamide, carboplatin, and etoposide versus gemcitabine and vinorelbine regimen for pediatric patients with relapsed or refractory Hodgkin's lymphoma. Front Oncol. (2023) 13:1153128. doi: 10.3389/fonc.2023.1153128. PMID: 37441423 PMC10335766

[69] Gokarn A Reddy L Hiregoudar S Poojary M Parab S Punatar S . A comparison of mobilization regimens used in Hodgkin lymphoma and factors that influence peripheral blood stem cell mobilization. Cytotherapy. (2025) 27:619–25. doi: 10.1016/j.jcyt.2025.01.004. PMID: 39903142

[70] Varma G Diefenbach C . The role of autologous stem-cell transplantation in classical Hodgkin lymphoma in the modern era. Semin Hematol. (2024) 61:253–62. doi: 10.1053/j.seminhematol.2024.06.003. PMID: 39039012

[71] Xiao A Mei M . Selection of a salvage regimen for relapsed or refractory classic Hodgkin lymphoma in the era of novel therapies. Hematol Oncol Clin North Am. (2026) 40(2):275–88. doi: 10.1016/j.hoc.2025.12.004. PMID: 41483984

[72] Kuruvilla J Ramchandren R Santoro A Paszkiewicz-Kozik E Gasiorowski R Johnson NA . Pembrolizumab versus brentuximab vedotin in relapsed or refractory classical Hodgkin lymphoma (KEYNOTE-204): An interim analysis of a multicentre, randomised, open-label, phase 3 study. Lancet Oncol. (2021) 22:512–24. doi: 10.1016/s1470-2045(21)00005-x. PMID: 33721562

[73] Jeffers L Santarsieri A Osborne W Chevillon F Vely A Bhartia V . Relapsed/refractory Hodgkin lymphoma after first-line escalated BEACOPDAC: Adverse outcomes are overcome by use of second-line targeted therapy - an international real-world analysis. Blood. (2025) 146:3623. doi: 10.1182/blood-2025-3623

[74] Kuruvilla J Modi D Santoro A Paszkiewicz-Kozik E Gasiorowski R Johnson NA . Pembrolizumab in relapsed or refractory Hodgkin lymphoma: A post hoc analysis of KEYNOTE-204 by prior lines of therapy. Leuk Lymphoma. (2025) 66:1710–9. doi: 10.1080/10428194.2025.2502805. PMID: 40548833

[75] Moskowitz AJ Shah G Schöder H Ganesan N Drill E Hancock H . Phase II trial of pembrolizumab plus gemcitabine, vinorelbine, and liposomal doxorubicin as second-line therapy for relapsed or refractory classical Hodgkin lymphoma. J Clin Oncol. (2021) 39:3109–17. doi: 10.1200/jco.21.01056. PMID: 34170745 PMC9851707

[76] Patel K Shah G Schoder H Ganesan N Drill E Hancock H . Pembrolizumab plus gemcitabine, vinorelbine, and liposomal doxorubicin as second-line therapy in relapsed or refractory Hodgkin lymphoma: 5-year update of a multicenter, phase 2 trial. Blood. (2025) 146:1850. doi: 10.1182/blood-2025-1850 40561302

[77] Daw S Cole PD Hoppe BS Hodgson D Beishuizen A Garnier N . Transplant-free approach in relapsed Hodgkin lymphoma in children, adolescents, and young adults: A nonrandomized clinical trial. JAMA Oncol. (2025) 11:249–57. doi: 10.1001/jamaoncol.2024.5627. PMID: 39745739 PMC11926625

[78] Harker-Murray P Mauz-Körholz C Leblanc T Mascarin M Michel G Cooper S . Nivolumab and brentuximab vedotin with or without bendamustine for R/R Hodgkin lymphoma in children, adolescents, and young adults. Blood. (2023) 141:2075–84. doi: 10.1182/blood.2022017118. PMID: 36564047 PMC10646780

[79] Forlenza CJ Gulati N Mauguen A Absalon MJ Castellino SM Franklin A . Combination brentuximab vedotin and bendamustine for pediatric patients with relapsed/refractory Hodgkin lymphoma. Blood Adv. (2021) 5:5519–24. doi: 10.1182/bloodadvances.2021005268. PMID: 34559223 PMC8714712

[80] LaCasce AS Bociek RG Sawas A Caimi P Agura E Matous J . Brentuximab vedotin plus bendamustine: A highly active first salvage regimen for relapsed or refractory Hodgkin lymphoma. Blood. (2018) 132:40–8. doi: 10.1182/blood-2017-11-815183. PMID: 29703778 PMC6073588

[81] Mei M Palmer J Lee HJ Isufi I Chen R Tsai NC . Nivolumab plus ifosfamide, carboplatin, and etoposide are a highly effective first salvage regimen in high-risk relapsed/refractory Hodgkin lymphoma. Hemasphere. (2025) 9:e70126. doi: 10.1002/hem3.70126. PMID: 40313510 PMC12042211

[82] Chen R Gopal AK Smith SE Ansell SM Rosenblatt JD Savage KJ . Five-year survival and durability results of brentuximab vedotin in patients with relapsed or refractory Hodgkin lymphoma. Blood. (2016) 128:1562–6. doi: 10.1182/blood-2016-02-699850. PMID: 27432875 PMC5034737

[83] Voorhees TJ McLaughlin EM Torka P Florindez J Kim NH Moyo TK . Outcomes in patients with classic Hodgkin lymphoma refractory or intolerant to brentuximab vedotin and anti-PD-1 therapy: A real world analysis from 15 U.S. academic centers. Blood Cancer J. (2025) 15:45. doi: 10.1038/s41408-025-01257-1. PMID: 40140364 PMC11947194

[84] Roemer MGM Redd RA Cader FZ Pak CJ Abdelrahman S Ouyang J . Major histocompatibility complex class II and programmed death ligand 1 expression predict outcome after programmed death 1 blockade in classic Hodgkin lymphoma. J Clin Oncol. (2018) 36:942–50. doi: 10.1200/jco.2017.77.3994. PMID: 29394125 PMC5877802

[85] Zak J Pratumchai I Marro BS Marquardt KL Zavareh RB Lairson LL . JAK inhibition enhances checkpoint blockade immunotherapy in patients with Hodgkin lymphoma. Science. (2024) 384:eade8520. doi: 10.1126/science.ade8520. PMID: 38900864 PMC11283877

[86] Benci JL Xu B Qiu Y Wu TJ Dada H Twyman-Saint Victor C . Tumor interferon signaling regulates a multigenic resistance program to immune checkpoint blockade. Cell. (2016) 167:1540–54.e12. doi: 10.1016/j.cell.2016.11.022. PMID: 27912061 PMC5385895

[87] Nie J Wang C Zheng L Liu Y Wang C Chang Y . Epigenetic agents plus anti-PD-1 reprogram the tumor microenvironment and restore antitumor efficacy in Hodgkin lymphoma. Blood. (2024) 144:1936–50. doi: 10.1182/blood.2024024487. PMID: 39093981

[88] Liu Y Wang C Li X Dong L Yang Q Chen M . Improved clinical outcome in a randomized phase II study of anti-PD-1 camrelizumab plus decitabine in relapsed/refractory Hodgkin lymphoma. J Immunother Cancer. (2021) 9(4):e002347. doi: 10.1136/jitc-2021-002347. PMID: 33820822 PMC8025784

[89] Liu Z Li X Gao Y Liu J Feng Y Liu Y . Epigenetic reprogramming of RUNX3 reinforces CD8 + T-cell function and improves the clinical response to immunotherapy. Mol Cancer. (2023) 22:84. doi: 10.1186/s12943-023-01768-0. PMID: 37189103 PMC10186650

[90] Wang C Liu Y Dong L Li X Yang Q Brock MV . Efficacy of decitabine plus anti-PD-1 camrelizumab in patients with Hodgkin lymphoma who progressed or relapsed after PD-1 blockade monotherapy. Clin Cancer Res. (2021) 27:2782–91. doi: 10.1158/1078-0432.Ccr-21-0133. PMID: 33674274

[91] Calabretta E Guidetti A Ricci F Di Trani M Monfrini C Magagnoli M . Chemotherapy after PD-1 inhibitors in relapsed/refractory Hodgkin lymphoma: Outcomes and clonal evolution dynamics. Br J Haematol. (2022) 198:82–92. doi: 10.1111/bjh.18183. PMID: 35468225 PMC9321573

[92] Liu X Zhang Q Guo H Pan Q Zhou K . Bendamustine, gemcitabine, and vinorelbine (BEGEV) regimen followed by ASCT induces durable remissions in PD-(L)1 inhibitor-resistant refractory/relapsed classical Hodgkin lymphoma: A single-center, long-term study. Blood. (2025) 146:1847. doi: 10.1182/blood-2025-1847 41642603

[93] Thiruvengadam S Poh C Lynch R Moskowitz C Spinner M Hutchings M . First-in-human, open-label, phase 1 study of a novel CD30-directed antibody-drug conjugate with a topoisomerase 1 inhibitor payload, PF-08046044 (35C), in patients with relapsed/refractory lymphomas: Updated safety, PK, preliminary efficacy and ctDNA analysis from dose escalation. Blood. (2025) 146:155. doi: 10.1182/blood-2025-155 40073374

[94] Nieto Y Banerjee P Kaur I Basar R Li Y Daher M . Allogeneic NK cells with a bispecific innate cell engager in refractory relapsed lymphoma: A phase 1 trial. Nat Med. (2025) 31:1987–93. doi: 10.1038/s41591-025-03640-8. PMID: 40186077 PMC13218020

[95] Wu J Fu J Zhang M Liu D . AFM13: A first-in-class tetravalent bispecific anti-CD30/CD16A antibody for NK cell-mediated immunotherapy. J Hematol Oncol. (2015) 8:96. doi: 10.1186/s13045-015-0188-3. PMID: 26231785 PMC4522136

[96] Sasse S Bröckelmann PJ Momotow J Plütschow A Hüttmann A Basara N . AFM13 in patients with relapsed or refractory classical Hodgkin lymphoma: Final results of an open-label, randomized, multicenter phase II trial. Leuk Lymphoma. (2022) 63:1871–8. doi: 10.1080/10428194.2022.2095623. PMID: 35848865

[97] Rothe A Sasse S Topp MS Eichenauer DA Hummel H Reiners KS . A phase 1 study of the bispecific anti-CD30/CD16A antibody construct AFM13 in patients with relapsed or refractory Hodgkin lymphoma. Blood. (2015) 125:4024–31. doi: 10.1182/blood-2014-12-614636. PMID: 25887777 PMC4528081

[98] Abdelhakim H Shouse G Kamdar M Pophali P Yamshon S Mountjoy L . Safety and efficacy of MT-601 in relapsed or refractory (R/R) Hodgkin lymphoma. Blood. (2025) 146:1846. doi: 10.1182/blood-2025-1846

[99] Gerrie AS Power MM Shepherd JD Savage KJ Sehn LH Connors JM . Chemoresistance can be overcome with high-dose chemotherapy and autologous stem-cell transplantation for relapsed and refractory Hodgkin lymphoma. Ann Oncol. (2014) 25:2218–23. doi: 10.1093/annonc/mdu387. PMID: 25149708

[100] Perales MA Ahmed S . When to use stem cell transplantation for classical Hodgkin lymphoma. Hematol Am Soc Hematol Educ Program. (2024) 2024:517–23. doi: 10.1182/hematology.2024000575. PMID: 39644064 PMC11665590

[101] Ahmed S Kumar A Carpenter P Herrera A Kelly K Pinnix C . American society of transplantation and cellular therapy clinical practice recommendations for transplantation in classical Hodgkin lymphoma. Transplant Cell Ther. (2026) 32:250–60. doi: 10.1016/j.jtct.2025.12.944. PMID: 41478324

[102] Alharthy H Ahmed S . The role of stem cell transplantation in Hodgkin lymphoma. Hematol Oncol Clin North Am. (2026) 40(2):289–301. doi: 10.1016/j.hoc.2025.12.003. PMID: 41549034

[103] Advani RH Moskowitz AJ Bartlett NL Vose JM Ramchandren R Feldman TA . Brentuximab vedotin in combination with nivolumab in relapsed or refractory Hodgkin lymphoma: 3-year study results. Blood. (2021) 138:427–38. doi: 10.1182/blood.2020009178. PMID: 33827139 PMC12684812

[104] Sureda A Pavlovsky A Haidar D Kristo F Stache V Zomas A . Real-world outcomes of brentuximab vedotin as consolidation therapy after autologous stem cell transplantation in relapsed/refractory Hodgkin lymphoma: A systematic review and meta-analysis. Bone Marrow Transplant. (2025) 60:820–31. doi: 10.1038/s41409-025-02557-7. PMID: 40200006 PMC12151861

[105] Moskowitz CH Walewski J Nademanee A Masszi T Agura E Holowiecki J . Five-year PFS from the AETHERA trial of brentuximab vedotin for Hodgkin lymphoma at high risk of progression or relapse. Blood. (2018) 132:2639–42. doi: 10.1182/blood-2018-07-861641. PMID: 30266774

[106] Herrera AF Chen L Nieto Y Holmberg L Johnston P Mei M . Brentuximab vedotin plus nivolumab after autologous haematopoietic stem-cell transplantation for adult patients with high-risk classic Hodgkin lymphoma: A multicentre, phase 2 trial. Lancet Haematol. (2023) 10:e14–23. doi: 10.1016/s2352-3026(22)00318-0. PMID: 36403579

[107] Liu P Wang C Sun P Yang H Wu M Nie M . Comparable survival with transplant-free approach versus autologous stem cell transplantation in adult relapsed/refractory classic Hodgkin lymphoma achieving complete remission. Blood. (2025) 146:5397. doi: 10.1182/blood-2025-5397

[108] Perales MA Awan FT Boumendil A Patel J Castagna L Angelucci E . Outcomes of allogeneic HCT in Hodgkin lymphoma in the era of checkpoint inhibitors: A joint CIBMTR and EBMT analysis. Blood. (2025) 146:1011–29. doi: 10.1182/blood.2024027197. PMID: 40623049 PMC12530899

[109] Faisal MS Hanel W Voorhees T Li R Huang Y Khan A . Outcomes associated with allogeneic hematopoietic stem cell transplantation for relapsed and refractory Hodgkin lymphoma in the era of novel agents. Cancer Med. (2023) 12:8228–37. doi: 10.1002/cam4.5631. PMID: 36653918 PMC10134314

[110] Sureda A Canals C Arranz R Caballero D Ribera JM Brune M . Allogeneic stem cell transplantation after reduced intensity conditioning in patients with relapsed or refractory Hodgkin’s lymphoma. Results of the HDR-ALLO study - a prospective clinical trial by the Grupo Español de Linfomas/Trasplante de Médula Osea (GEL/TAMO) and the Lymphoma Working Party of the European Group for Blood and Marrow Transplantation. Haematologica. (2012) 97:310–7. doi: 10.3324/haematol.2011.045757. PMID: 21993674 PMC3269494

[111] Mariotti J Pinton C Giordano L Taurino D Sarina B De Philippis C . Reduced incidence of relapse after checkpoint inhibitors relative to brentuximab vedotin as salvage therapy before allogeneic stem cell transplantation for refractory/relapsed Hodgkin lymphoma: A retrospective analysis. Br J Haematol. (2025) 207:1684–9. doi: 10.1111/bjh.70091. PMID: 40887735 PMC12512095

[112] Koca O Eskazan AE . Refining the risk stratification in advanced-stage classical Hodgkin lymphoma: A critical analysis of clinical prediction models. Br J Haematol. (2025) 207:1744–54. doi: 10.1111/bjh.70115. PMID: 40843643 PMC12624167

[113] Rodday AM Parsons SK Upshaw JN Friedberg JW Gallamini A Hawkes E . The advanced-stage Hodgkin lymphoma international prognostic index: Development and validation of a clinical prediction model from the Holistic Consortium. J Clin Oncol. (2023) 41:2076–86. doi: 10.1200/jco.22.02473. PMID: 36495588 PMC10082254

[114] Cui Z Rodday AM Tighiouart H Counsell N Rossetti S Upshaw J . PET-adaptive BEACOPP- versus ABVD-based therapies for advanced-stage (AS) classic Hodgkin lymphoma (CHL): Survival comparisons leveraging a multi-state model and analyzing the impact of A-HIPI scores across the disease course. Blood. (2025) 146:129. doi: 10.1182/blood-2025-129 40638203

[115] Rodday AM Evens AM Maurer MJ Upshaw JN Counsell N Rossetti S . An individualized prediction model for early-stage classic Hodgkin's lymphoma. NEJM Evid. (2025) 4:EVIDoa2500115. doi: 10.1056/EVIDoa2500115. PMID: 40536772 PMC12498259

[116] Rodday AM Eichenauer D Jablonski J Borchmann S Tighiouart H Upshaw J . External validation of the early-stage classic Hodgkin lymphoma (CHL) international prognostication index (E-HIPI) in the German Hodgkin Study Group (GHSG) unfavorable clinical trials. Blood. (2025) 146:1845. doi: 10.1182/blood-2025-1845

[117] Shahsavand A Forghani S Kharaghani MA Samiee R Mekary RA Aliasghari MM . Prognostic utility of circulating tumor DNA in classical Hodgkin lymphoma: A systematic review and individual participant data Bayesian meta-analysis. Crit Rev Oncol Hematol. (2026) 219:105154. doi: 10.1016/j.critrevonc.2026.105154. PMID: 41581737

[118] Paczkowska J Kaszkowiak M Leblanc M Stewart C Herrera A Fernandez del Castillo JC . Circulating tumor DNA analyses of molecular tumor burden are superior to PET-assessed responses in patients with advanced stage classic Hodgkin lymphoma treated on SWOG S1826. Blood. (2025) 146:351. doi: 10.1182/blood-2025-351

[119] Heger JM Mattlener J Kaul H Ferdinandus J Schneider J Schleifenbaum JK . MRD-2 in the GHSG HD21 trial assessed by a validated circulating tumor DNA sequencing assay. Blood. (2026) 147(19):2194–202. doi: 10.1182/blood.2025031089. PMID: 41662627

[120] Plattel WJ Visser L Diepstra A Glaudemans A Nijland M van Meerten T . Interim thymus and activation regulated chemokine versus interim (18) F-fluorodeoxyglucose positron-emission tomography in classical Hodgkin lymphoma response evaluation. Br J Haematol. (2020) 190:40–4. doi: 10.1111/bjh.16514. PMID: 32106342 PMC7383815

[121] Teesink SA Visser L Nijland M Keijzer K Niezink AGH Kroesen BJ . Serum TARC: A biomarker for early detection or exclusion of relapse in classic Hodgkin lymphoma. Blood Adv. (2025) 9:4618–21. doi: 10.1182/bloodadvances.2025015837. PMID: 40540801 PMC12455077

[122] Toner K Renfro LA Dave H Pezzella G Pei Q Giulino-Roth L . Tumor-specific immune responses and biomarkers in pediatric patients with high-risk Hodgkin lymphoma. Blood Adv. (2026) 10:183–91. doi: 10.1182/bloodadvances.2025016797. PMID: 40990939 PMC12805261

[123] Husi K Pinczés LI Fejes Z Nagy B Illés Á Miltényi Z . Combined prognostic role of TARC and interim (18)F-FDG PET/CT in patients with Hodgkin lymphoma-real world observational study. Hell J Nucl Med. (2022) 25:125–31. doi: 10.1967/s002449912471. PMID: 35913858

[124] Kredátusová A Chupáň T Móciková H Sýkorová A Marková J Lukášová M . Soluble cytokines enhance risk prediction across all stages of classical Hodgkin lymphoma. biomark Res. (2026) 14:14. doi: 10.1186/s40364-025-00881-0. PMID: 41540469 PMC12809970

[125] Levin LI Breen EC Birmann BM Batista JL Magpantay LI Li Y . Elevated serum levels of SCD30 and IL6 and detectable IL10 precede classical Hodgkin lymphoma diagnosis. Cancer Epidemiol Biomarkers Prev. (2017) 26:1114–23. doi: 10.1158/1055-9965.Epi-16-1012. PMID: 28341757 PMC5511544

[126] Yılmaz U Birtaş Ateşoğlu E Ferhanoğlu B . Tight competition for the first-line treatment of Hodgkin’s lymphoma: A comprehensive review of practice-changing developments in recent years. Expert Opin Biol Ther. (2025) 25:731–47. doi: 10.1080/14712598.2025.2519524. PMID: 40506833

[127] Burton C Allen P Herrera AF . Paradigm shifts in Hodgkin lymphoma treatment: From frontline therapies to relapsed disease. Am Soc Clin Oncol Educ Book. (2024) 44:e433502. doi: 10.1200/edbk_433502. PMID: 38728605

[128] Evens AM . Newly diagnosed classical Hodgkin lymphoma: Optimizing outcomes in a new therapeutic era. Hematol Oncol. (2025) 43 Suppl 2:e70066. doi: 10.1002/hon.70066. PMID: 40517538 PMC12167649

[129] Alibrahim MN Carbone A Alsaleh N Gloghini A . Immune checkpoint molecules in Hodgkin lymphoma and other hematological Malignancies. Cancers (Basel). (2025) 17(14):2292. doi: 10.3390/cancers17142292. PMID: 40723175 PMC12293259

[130] Velasco R Domingo-Domenech E Sureda A . Brentuximab-induced peripheral neurotoxicity: A multidisciplinary approach to manage an emerging challenge in Hodgkin lymphoma therapy. Cancers (Basel). (2021) 13(23):6125. doi: 10.3390/cancers13236125. PMID: 34885234 PMC8656789

[131] Fattore D Lauletta G Pages C Theret V Sibaud V . Update on dermatological toxicities of immune checkpoint inhibitors. Presse Med. (2025) 55:104330. doi: 10.1016/j.lpm.2025.104330. PMID: 41285238

[132] Tanase-Damian C Antone NZ Paun DL Tanase I Achimaș-Cadariu PA . Reproductive toxicity of immune checkpoint inhibitors in triple-negative breast cancer: A case report with a literature review. Diseases. (2026) 14(2):51. doi: 10.3390/diseases14020051. PMID: 41745089 PMC12939086

[133] Hewamana S Kandabadage L Skandarajah T Peiris N Abeyaratne S Arseculeratne G . Applicability of protocols from high-income countries in a resource limited setting; real world data of histopathology, clinical features and long-term outcome of Hodgkin lymphoma in Sri Lanka. EClinicalMedicine. (2021) 38:100998. doi: 10.1016/j.eclinm.2021.100998. PMID: 34278283 PMC8267551

[134] Fu L Zhou X Zhang X Li X Zhang F Gu H . Circulating tumor DNA in lymphoma: Technologies and applications. J Hematol Oncol. (2025) 18:29. doi: 10.1186/s13045-025-01673-7. PMID: 40069858 PMC11900646

[135] Kearney M Faithfull S Leech M . From cure to care-understanding long-term health risks after Hodgkin's lymphoma treatment in adolescents and young adults. Int J Adolesc Med Health. (2025) 38(2):103–10. doi: 10.1515/ijamh-2025-0131. PMID: 41267326

